# Large-scale RNAi screens identify novel genes that interact with the *C. elegans *retinoblastoma pathway as well as splicing-related components with synMuv B activity

**DOI:** 10.1186/1471-213X-7-30

**Published:** 2007-04-06

**Authors:** Julian Ceron, Jean-François Rual, Abha Chandra, Denis Dupuy, Marc Vidal, Sander van den Heuvel

**Affiliations:** 1 Massachusetts General Hospital Cancer Center and Harvard Medical School, Building 149, 13th Street, Charlestown, 02129 MA, USA; 2 Center for Cancer Systems Biology (CCSB) and Department of Cancer Biology, Dana-Farber Cancer Institute, and Department of Genetics, Harvard Medical School, 44 Binney Street, Boston, 02115 MA, USA; 3 Department of Developmental Biology, Utrecht University, Kruytbuilding, Padualaan 8, 3584 CH Utrecht, The Netherlands; 4 Present address : Molecular Oncology and Aging Research, Centre d'Investigacions en Bioquímica I Biología Molecular (CIBBIM), Hospital Universitari Vall d'Hebron 119-129, Barcelona 08035, Spain

## Abstract

**Background:**

The *retinoblastoma *tumor suppressor (Rb) acts in a conserved pathway that is deregulated in most human cancers. Inactivation of the single Rb-related gene in *Caenorhabditis elegans, lin-35*, has only limited effects on viability and fertility, yet causes changes in cell-fate and cell-cycle regulation when combined with inactivation of specific other genes. For instance, *lin-35 *Rb is a synthetic multivulva (synMuv) class B gene, which causes a multivulva phenotype when inactivated simultaneously with a class A or C synMuv gene.

**Results:**

We used the ORFeome RNAi library to identify genes that interact with *C. elegans lin-35 *Rb and identified 57 genes that showed synthetic or enhanced RNAi phenotypes in *lin-35 *mutants as compared to *rrf-3 *and *eri-1 *RNAi hypersensitive mutants. Based on characterizations of a deletion allele, the synthetic *lin-35 *interactor *zfp-2 *was found to suppress RNAi and to cooperate with *lin-35 *Rb in somatic gonad development. Interestingly, ten splicing-related genes were found to function similar to *lin-35 *Rb, as synMuv B genes that prevent inappropriate vulval induction. Partial inactivation of specific spliceosome components revealed further similarities with *lin-35 *Rb functions in cell-cycle control, transgene expression and restricted expression of germline granules.

**Conclusion:**

We identified an extensive series of candidate *lin-35 *Rb interacting genes and validated *zfp-2 *as a novel *lin-35 *synthetic lethal gene. In addition, we observed a novel role for a subset of splicing components in *lin-35 *Rb-controlled processes. Our data support novel hypotheses about possibilities for anti-cancer therapies and multilevel regulation of gene expression.

## Background

The retinoblastoma gene (Rb) was the first tumor suppressor gene to be genetically identified and cloned, based on germline mutations in familial cases of retinoblastoma [1]. Since, deregulation of the Rb pathway has been found to be a general aspect of a wide variety of human cancers, with ~80% of all sporadic cancers containing alterations in Rb or its regulatory components [2,3]. The frequency of mutations in the Rb gene itself varies greatly between different tumor types, which likely relates to expression levels of other members of the Rb family (p130 and p107) that are closely related in structure and show partly redundant functions *in vivo *[4].

Proteins of the pRb family function as transcriptional repressors to control cell division, differentiation and death [[5], and citations herein]. To control cell proliferation, pRb family members inhibit transcription of S phase promoting genes by binding and blocking activating E2F transcription factors (in mammals: E2F-1, E2F-2 or E2F-3 in association with DP-1 or DP-2). Another group of E2F transcription factors acts together with the pRb protein family to repress transcription, including heterodimers of mammalian E2F-4 or E2F-5 and one of the two DP proteins [6]. Besides E2F family members, pRb-related proteins have been reported to physically interact with a large number of other cellular proteins, including components of chromatin remodeling complexes [e.g.: [7,8]]. Although the physiological relevance of many interactions remains to be determined, the functions of pRb likely involve a variety of different protein complexes that either block activation of transcription, or actively repress transcription through recruitment of histone-modification and chromatin-remodeling complexes.

The gene *lin-35 *encodes the single *Caenorhabditis elegans *homolog of the pRb family. Animals with putative null mutations in *lin-35 *are viable and fertile [9], although homozygous mutants show a reduced brood size, delayed development, and larval arrest with low penetrance [[10,11] and our unpublished observations]. Previous studies revealed redundant functions of *lin-35 *in a variety of processes such as vulva formation, cell-cycle control, development of the pharynx and somatic gonad, larval development, distal tip cell (DTC) migration and meiotic progression [9-21].

The first and best-characterized function of *lin-35 *is inhibition of the vulval cell fate [[9] and references herein]. In this process, *lin-35 *acts as a synthetic Multivulva (synMuv) class B gene redundantly with class A and C genes [20]. Only animals that contain mutations in two different classes, e.g. A and B, display a Multivulva phenotype (a phenotype that depends on two simultaneous mutations is referred to as "synthetic", hence synMuv). Several synMuv B genes act also in cell-cycle control [12,22] and many encode homologs of well-known partners of pRb, including *efl-1 *E2F and *dpl-1 *DP [23]. Biochemical purification of Rb complexes revealed extensive overlap between components of the *Drosophila *myb/dREAM complex and *C. elegans *synMuv B proteins [6,24]. Together, these data highlight the evolutionary conservation of the Rb pathway as well as the valuable contributions of synMuv B mutants in identifying functional partners of Rb family members.

Since *lin-35 *is a non-essential gene and its functions are redundant, we decided to perform a synthetic screen to identify additional genes that interact with *lin-35 *Rb. This screen aimed to identify genes whose functions are essential specifically when *lin-35 *Rb is inactive. As the Rb pathway is generally compromised in tumor cells, this strategy has the potential of identifying targets for anticancer drugs, because inactivating a gene that is synthetic lethal with loss of Rb activity could preferentially reduce the viability of cancer cells. In a large-scale RNAi approach based on the ORFeome RNAi library [25], we identified 57 *lin-35 *interacting genes. In addition, we found a novel group of synMuv B genes that encode components of the splicing machinery. Thus, our screens have revealed many novel genes that may act in parallel or in a linear pathway with *lin-35 *Rb, some of which may be followed as candidate targets for cancer therapies.

## Results and discussion

To expand on previous studies of synthetic interactions with *lin-35 *Rb, we used RNAi by feeding to inhibit the function of 10,953 genes present in the ORFeome RNAi library v1.1. We compared the effects of feeding RNAi in wild-type (WT) animals and *lin-35(n2239) *Rb candidate "null" mutants [9]. In the initial screen, we identified 523 genes that showed a variety of synthetic or enhanced phenotypes in *lin-35(n2239) *as compared to WT (N2) animals (see: Methods; Additional file 1, Supplementary Table 1A; for legend, see Additional file 6).

*lin-35 *Rb and several other genes that act with Rb in transcriptional repression and chromatin remodeling in other species have been identified in *C. elegans *based on their inhibitory role in vulval cell-fate specification. Several strains deficient for these so-called synMuv B genes, including *lin-35 *mutants, were recently found to be RNAi hypersensitive [26]. To exclude candidates that show a non-specific stronger RNAi phenotype in *lin-35(n2239)*, we included the non-Rb related RNAi hypersensitive strain *rrf-3(pk1426) *in a secondary screen [27]. This assay removed 279 initially selected genes, as they showed similar RNAi phenotypes in *rrf-3 *and *lin-35 *mutants. However, for 244 candidate genes, we reproducibly observed a stronger feeding RNAi phenotype in *lin-35(n2239) *than in *rrf-3(pk1426) *and WT animals (Supplementary Table 1A). The majority of genes (50/69) for which no RNAi phenotype had been previously reported belonged to this group (according to wormbase version WS153 [66], see Additional file 1, Supplementary Table 1A).

To further validate the specificity of the interactions with *lin-35 *Rb, we tested the 244 candidate genes in a more extensive panel of RNAi hypersensitive strains, including *rrf-3*(*pk1426*), *eri-1(mg366)*, the synMuv B mutants *hpl-2*(*ok917*), *lin-15B*(*n744*), *lin-35*(*n2339*) as well as *lin-37*(*n758*), and double mutant *eri-1*(*mg366*); *lin-15B*(*n744*) animals. In agreement with Cui *et al *[28] but not Wang *et al *[26], we observed that *lin-37(n758) *synMuv B animals are RNAi hypersensitive (Table 1). The strain *eri-1*; *lin-15B *was included as an extreme RNAi hypersensitive control, with the caveat that this strain is somewhat sick and was found to display a variety of defects with low penetrance (data not shown).

**Table 1:** Novel RNAi hypersensitive strains and gene-dependent hypersensitivity

		**N2**	***rrf-3 (pk1426)***	***lin-35 (n2239)***	***lin-37 (n758)***	***zfp-2 (tm557)***
**RNAi**	**Phenotype**					

*gpc-2*	Emb	0%	14.9%	2.6%	2.8%	0%
*mom-2*	Emb	17.8%	40.1%	64.1%	67.4%	69.4%
*hmr-1*	Lvl	0%	Ste, Ooc, Emb	80.7%	77.6%	53.1%
*cel-1*	Lva	0.5%	100%	98.6%	11%*	23.2%
*unc-87*	Unc	3.6%	0%	94.2%	85.3%	0%
*unc-15*	Unc	0%	100%	96.5%	92.6%	0%

We tested several RNAi clones, previously used by others to quantitatively examine sensitivity to RNAi, in different genetic backgrounds [26,62,63]. To allow comparison with RNAi effects reported by others, we used RNAi clones from the Ahringer Library [64] except for *cel-1*(Vidal library). Numbers are averages of 3 to 6 experiments and indicate proportion of animals (F1 progeny) displaying the corresponding phenotype. In agreement with recent findings [26,65], we also observed that different RNAi hypersensitive mutants showed surprising gene specificity in RNAi enhancement.

*cel-1 RNAi in *lin-37*(*n758*) produces larval arrest at later stages

**Figure 1:**
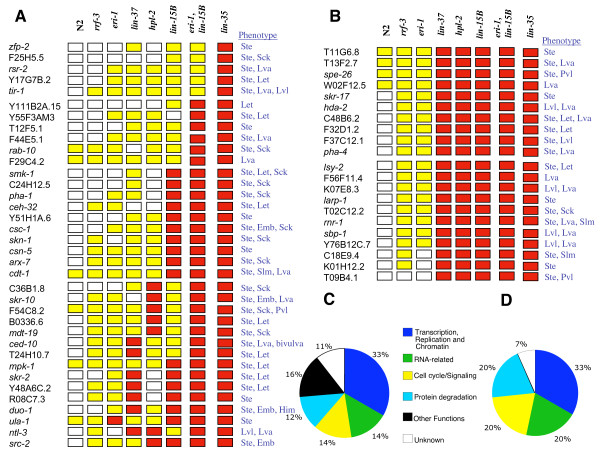
(A, B) Color code representation of the strength of RNAi phenotypes of candidate genes in different genetic backgrounds. Red indicates a highly penetrant lethal phenotype. Yellow designates a substantially weaker phenotype and/or increase in viable progeny and white indicates weak or no detectable defects. (A) Synthetic lethal or enhanced RNAi phenotypes. (B) RNAi phenotypes enhanced in synMuv B mutants See: Additional file 1, Supplementary Table 1, for details. (C) Pie Chart representing the major functional categories among the 57 candidates genes (See Additional file 1, Supplementary Table 1C). (D) Pie Chart representing major functional categories among 14 genetic interactors previously published (See Additional file 3, Supplementary Figure 1).

In the combined screens, we identified 57 genes whose RNAi phenotype was reproducibly stronger in *lin-35*, or in multiple synMuv B mutants, than in WT, *rrf-3 *and *eri-1*. These 57 genes can be classified in three groups: 5 genes showed an RNAi phenotype substantially stronger in *lin-35(n2239) *than in any other background, 31 genes displayed strong RNAi phenotypes in *lin-35(n2239) *and in one or two other synMuv B mutants, and 21 genes showed strong RNAi phenotypes in all synMuv B mutants examined (Figure 1) (see Additional file 1, Supplementary Table 1B). We consider all three classes of interest, as synthetic interactions could either be specific for *lin-35 *or take place with multiple Rb-pathway components. The group of 31 candidates includes *pha-1*, which was previously shown to interact with *lin-35 *[14] and provides a first validation that our approach identified specific interactions. In addition, the various classes of genes identified are in agreement with previous genetic studies that revealed redundant functions of *lin-35 *in various developmental processes [9-21]. A high percentage of the genes identified are predicted to fulfill roles in transcription, DNA replication and chromatin structure, as was expected for genes that act redundantly with *lin-35 *Rb (Figure 1C–D). Genes involved in protein degradation were also expected to interact with the *lin-35 *transcriptional repressor. Such genes, as well as genes involved in signaling, cell cycle and RNA metabolism, were all well represented in our screens as in previous screens (Fig. 1C–D, see Additional file 3).

Recently, Lehner *et al*. described the existence of "hub" genes. This group of genes encodes chromatin regulators that modify diverse signaling pathways and therefore genetically interact with many other genes [29]. As *lin-35 *is expected to function in chromatin remodeling and interacted with a large number of genes in our screen, we suggest that Rb is one of such "hub" genes.

Importantly, redundant (synthetic) interactions with *lin-35 *Rb have often been revealed through partial loss-of-function mutations, while complete loss of function of the same genes caused lethality (e.g.: *pha-1 *[14], *psa-1 *[16], *him-17 *[18] and *gon-14 *[20]; the class A synMuv genes are a clear exception). Consistent with this notion, we injected dsRNA to achieve strong RNAi phenotypes for a sample of 12 candidate genes (including *pha-1*, the known *lin-35 *interactor), and all but *zfp-2 *caused lethality/sterility with or without co-injection of *lin-35 *dsRNA (see Additional file 1, Supplementary Table 1B). Therefore, synthetic interaction with *lin-35 *Rb may be detected with partial loss-of-function alleles (or by feeding dsRNA) but not in a setting approaching strong loss-of-function or null conditions (as may be achieved by injecting dsRNA). Thus, independent validation of these genetic interactions will require appropriate alleles.

Similarly, a feeding RNAi phenotype in an *eri-1 *or *rrf-3 *mutant background does not rule out genetic interaction with *lin-35 *Rb. However, genes that show an apparent feeding RNAi phenotype only in *lin-35 *mutants, and not even in *eri-1; lin-15B *RNAi hypersensitive mutants, are the best candidates for a functional interaction with *lin-35 *Rb. Only five genes showed this level of specificity: *zfp-2*, F25H5.5, *rsr-2*, Y17G7B.2 and *tir-1*.

F25H5.5 encodes the putative *C. elegans *homolog of Claspin, which in vertebrates mediates activation of the Chk1 kinase as part of a DNA replication checkpoint response [30]. As pRb and Claspin both negatively regulate cell-cycle progression, a synthetic lethal interaction is conceivable. It will be attractive to examine whether reducing human Claspin activity specifically increases the killing of Rb-minus tumor cells, if not alone then possibly in response to e.g. gamma-irradiation (experiments in progress).

The predicted Y17G7B.2 product is similar to the *trithorax *group protein Ash2, a subunit of a histone H3 (Lys4) methyltransferase complex [31]. Genetic interaction between the Ash2 and pRb family members is plausible, as both form part of protein complexes that regulate gene expression through histone modification, and physical interactions between components of these complexes have been reported [32]. Indeed, others recently observed a genetic interaction between mutant alleles of Y17G7B.2/*ash-2 *and *lin-35 *(G. Soete and HC Korswagen, manuscript in preparation), which validates the identification of this gene in our screen.

The *tir-1 *gene encodes a protein with a conserved Toll/IL-1 resistance (TIR) domain and has been shown to act in a conserved MAP kinase pathway in innate immunity [33,34] as well as lateral signaling between specific neurons [35]. The basis of a genetic interaction between *tir-1 *and *lin-35 *Rb is not immediately obvious. However, if confirmed it should be of considerable interest, as TIR-1 contains a single human ortholog, SARM. Like TIR-1, SARM contains a TIR domain, heat-armadillo repeats and two sterile alpha motifs (SAM) [35].

The *zfp-2 *gene encodes a zinc-finger transcription factor with six C2H2 zinc finger domains and a KRAB A domain, which is associated with transcriptional repression [36] (see Additional file 4). Human pRb has been reported to physically interact with two zinc finger proteins named RIZ1 and RBak. RIZ1 is commonly inactivated in human cancers, and RBak contains a KRAB domain and has homology with *zfp-2 *[37-39]. Finally, *rsr-2 *encodes a serine (S) and arginine (R)-rich protein with homology to the human splicing co-activator Srm300. The *zfp-2 *and *rsr-2 *genes and their interactions with *lin-35 *are discussed below.

### The zinc finger *zfp-2 *cooperates with *lin-35 *in somatic gonad development

Of the five candidates that interacted specifically with *lin-35 *(Figure 1A, top), viable mutations were only available for *zfp-2 *and *tir-1*. We did not observe a synthetic interaction between *lin-35 *and the *tir-1 *deletion alleles *ok1052 *and *tm1111*. However, these alleles likely do not cause *tir-1 *inactivation, as the deletions affect specific splice forms while two of the confirmed *tir-1 *mRNAs (F13B10.1b and d) [66] should be expressed normally. In contrast, we observed a strong synthetic interaction between the *zfp-2 *allele *tm557 *and the *lin-35 *candidate null mutations *n745 *and *n2239 *as well as *lin-35(RNAi)*. The *zfp-2*(*tm557) *mutation is a 233 bp deletion which removes 130 bp of coding sequences and changes the reading frame shortly after the predicted translation-initiation codon.

**Figure 2:**
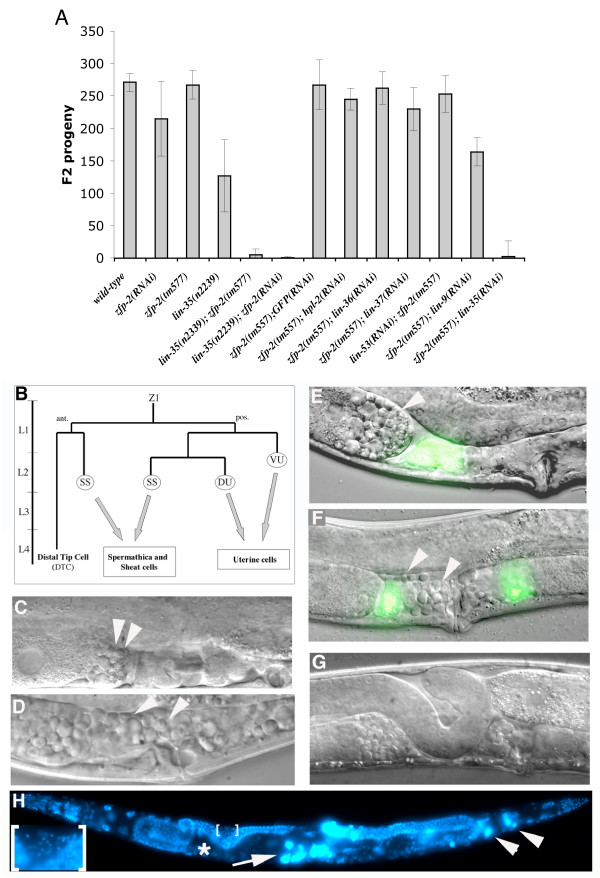
**Genetic interaction between *zfp-2 *and *lin-35 *Rb**. (A) Double inactivation of *zfp-2 *and *lin-35 *causes sterility. Total numbers of progeny were counted for 77 single *lin-35*(*n2239*); *zfp-2*(*tm557*) hermaphrodites. For other genotypes, total progeny of 6 to 12 single hermaphrodites were counted. Error bars indicate standard deviation.(B-H) Somatic gonad defects in animals deficient for *zfp-2 *and *lin-35*. (B) Simplified scheme representing the lineage of Z1 through larval stages, which it is one of the two precursors of the somatic gonad. (ant: anterior, post: posterior, SS: sheath/spermathecal precursor, DU and VU: dorsal and ventral uterine precursors). (C) Detail of WT proximal hermaphrodite gonad. White arrowheads point to spermatocytes and spermatids in panels C-F. (D) Detail of sterile *lin-35*(*n2239*); *zfp-2*(*tm557*) adult hermaphrodite with defective spermatheca and uterus. Some sperm precursors, spermatocytes and spermatids, are misplaced. (E) Expression of *fkh-6*::GFP in *lin-35 *(RNAi) animals. Sperm precursors are properly located. (F) Expression of *fkh-6*::GFP in *lin-35(RNAi); zfp-2*(*tm557*) animals. Sperm precursors are misplaced. Expression of *fkh-6 *is required for normal differentiation of the spermatheca and uterus [60] and appears normal, indicating that cell specification is initiated. (G) Defect in Distal Tip Cell migration in *lin-*35(*n2239*); *zfp-2(tm557*) animal. (H) *lin-35(n2239); zfp-2(tm557) *adult, DNA stained with DAPI. Note aberrant turn of one gonad arm (*), endomitotic oocytes (Emo) (arrow) and polyploid somatic nuclei (arrowheads). Proper proximal gonad structures such as spermatheca are missing. Lower left: High magnification of the bracketed area. Note misplaced sperm (small and intense blue spots).

Inactivation of *zfp-2 *alone by RNAi (feeding or injection) or the *zfp-2*(*tm557*) homozygous deletion did not cause any apparent abnormalities (Figure 2A,C). Although *lin-35 *mutants are also viable, their brood size is reduced (Figure 2A and [10]). We observed a variety of defects in *lin-35(n2239) *mutants with low penetrance, including reduced integrity of the proximal gonad (uterus and spermatheca), a low percentage of endomitotic oocytes (Emo), DTC migration defects (8.4%, n = 107) and abnormalities in vulval morphology (3.7 % everted vulva, n = 107). Loss-of-*lin-35 *function also allowed continued nuclear division of intestinal nuclei during larval development (variable and partly dependent on developmental stage and temperature, ranging between normal (32–34 nuclei) and 55 nuclei/animal [[17,19], and our unpublished observations].

Importantly, *lin-35*(*n2239*); *zfp-2(tm557) *double mutants were almost completely sterile (Figure 2A). In addition, defects in gonad migration (18% n = 188) and vulval morphology (17% n = 188) were enhanced compared to *lin-35 *alone and polyploid nuclei were seen in the soma (Figure 2H). Such sterile double mutant animals showed dramatic morphological defects in the proximal somatic gonad, with absence of a normal uterine cavity and, based on misplaced sperm precursors and spermatids, dysfunctional spermatheca (Figure 2D,2F and 2H). Oocytes were present but in general did not ovulate, were not fertilized and often became polyploid (endomitotic oocytes: Emo phenotype [40]) (Figure 2H). These defects resemble phenotypes observed after ablation of somatic cell precursors in the germ line, which indicated that sheath and spermathecal cells are required for ovulation and fertilization [41].

**Figure 3:**
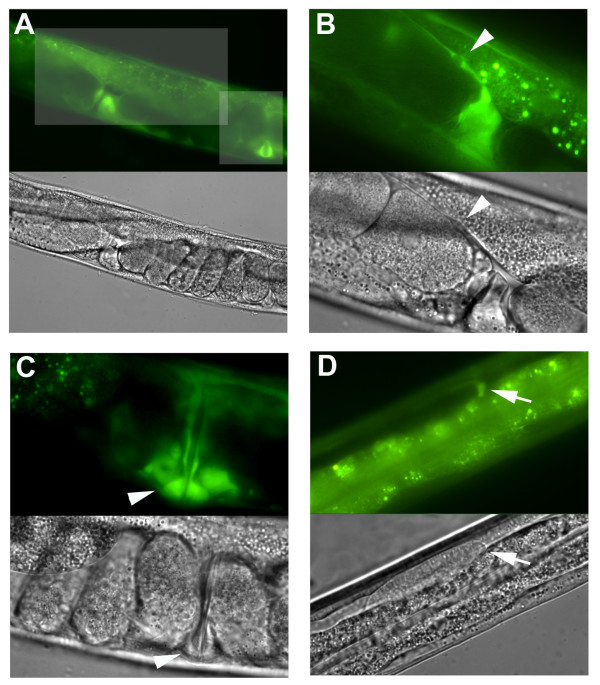
***zfp-2*::GFP is expressed in the somatic gonad**. (A)* zfp-2*::GFP expression in somatic gonad tissue and vulval cells. (B) Magnification of the rectangular area highlighted in A. GFP expression in spermatheca and sheath cells. Arrowhead indicates sheath cell. (C) Magnification of the square highlighted in A. GFP expression in several vulval cells. Arrowheads indicate equivalent vulval cells (D) GFP expression in distal tip cell (DTC) (arrow). In addition, we observed GFP expression in some neurons, pharyngeal cells, intestine and tail (not shown). The corresponding light microscopy images are shown below epifluorescence figures.

Examination of the *zfp-2 *expression pattern suggested that the affected tissues express both *lin-35 *and *zfp-2*. While *lin-35 *Rb seems to be ubiquitously expressed [9,42], *zfp-2 *expression is more restricted. Transgenic animals expressing the Green Fluorescent Protein (GFP) under the control of the *zfp-2 *promoter showed fluorescence in vulval cells and all somatic gonad structures such as spermatheca, sheath cells, uterine cells and distal tip cells (DTCs) (Figure 3). This expression pattern correlates well with the observed synthetic phenotype. Together, these observations indicate that *lin-35 *and *zfp-2 *function redundantly in development of the somatic gonad lineages derived from Z1 and Z4 (Figure 2B: DTCs, spermatheca, sheath cells and uterine cells)[43]. This may explain the abnormal migration of DTCs, morphological defects of the uterus and spermatheca, and synthetic sterile phenotype of *lin-35*(*n2239*); *zfp-2(tm557) *double mutants.

Although gonadal defects were apparent, the developmental program is partially completed and cell specification, based on reporter gene expression and structure, does occur (Figure 2F). Some of the sheath cells were apparently missing in the double mutants, but quantitative analysis was hampered by silencing of the sheath cell marker (*lim-7*::GFP [44]) upon injection of *lin-35 *and *zfp-2 *dsRNA (Supplementary Figure 3). Knock down of *lin-35 *and *zfp-2 *after completion of somatic gonad development did not cause sterility. Specifically, when *lin-35 *and *zfp-2 *dsRNA were injected in the gonad of L4 larvae or young adult animals, the injected animals remained fertile while their progeny were sterile. Although such experiments do not provide strong evidence, the results are consistent with redundant functions for *lin-35 *and *zfp-2 *in somatic gonad development, rather than oocyte maturation.

F35H8.3*/zfp-2 *was previously found in an RNAi screen as one of 59 genes required for cosuppression [45]. Cosuppression refers to the coincident silencing of a repetitive transgene and corresponding endogenous gene, which involves chromatin remodeling and RNAi pathway components [45]. Remarkably, we observed that *zfp-2*(*tm557*) mutants are not RNAi resistant but hypersensitive to RNAi for certain genes (Table 1). Thus, *zfp-2 *and *lin-35 *both suppress RNAi, which indicates a more extensive functional relationship. As yet another similarity with *lin-35*, *zfp-2 *RNAi also caused silencing of the *rol-6 *transgene (see Additional file 5). Based on the similarities and genetic interaction with *lin-35*, we favor the model that ZFP-2 acts as a zinc-finger transcription factor in transcriptional suppression.

In conclusion, *zfp-2 *and *lin-35 *show a synthetic lethal interaction and cooperate in development of the somatic gonad, likely acting in parallel or together in transcription repression and/or chromatin remodeling.

### Knock down of specific splicing components resembles the *lin-35 *Rb phenotype

The synMuv B gene *lin-35 *Rb represses ectopic induction of the vulval cell fate redundantly with synMuv A and C genes. We did not observe synthetic Multivulva phenotypes in our screen, not even for the two known class A genes or single class C gene present in the library. This fits with our observations that class A genes tend to score false negative in feeding RNAi screens (our unpublished results).

Although synthetic interaction between null alleles often points to functions in parallel pathways, several observations indicate that class A and B synMuv proteins could in fact act in conjunction [46,47]. Similarly, genetic interaction with *lin-35 *Rb in our screen could reveal genes that act in parallel pathways, but possibly also identify additional components of a LIN-35 protein complex or pathway. As such, it was of interest that a known synMuv B gene, *mep-1*, was found in the set of 244 candidates. To examine whether additional synMuv B genes were present among the putative *lin-35 *interactors, we performed feeding RNAi for the 244 candidates in the synMuv A mutant *lin-15A*(*n767*). This screen identified *rsr-2*, one of the five genes that showed a feeding RNAi phenotype specifically in *lin-35 *mutants (Figure 1A), as a novel synMuv B gene.

Despite the *lin-35 *specific feeding RNAi phenotype, *rsr-2 *dsRNA injection caused severe abnormalities such as sterility, embryonic lethality and larval arrest [[48], and this report]. Interestingly, *rsr-2 *encodes a serine (S) and arginine (R)-rich protein with homology to the human splicing co-activator Srm300, which acts with other splicing factors to perform its functions. To test whether additional splicing factors belong to the synMuv B family, we collected feeding RNAi clones corresponding to 135 putative *C. elegans *splicing-related genes (see Additional file 2, Supplementary Table 2A; legend, see Additional file 6). We assayed these 135 genes by feeding RNAi in L1 and young adult *lin-15A *mutants (see Additional file 2, Supplementary Table 2B). An additional nine genes showed a Muv phenotype in *lin-15A *and other class A mutants (Table 2), but not in wild-type or class B animals, classifying these genes as synMuv B. These nine genes include the seven members of the Sm family in *C. elegans *(*snr-1, 2, 3, 4, 5, 6 *and *7*) [49] as well as two Sm-like genes (*lsm-2/gut-2 *and *lsm-4*), all of which are well conserved through evolution. Only *lsm-2 *and *lsm-4 *RNAi produced viable animals at 25°C, at which temperature the synMuv phenotype is most highly penetrant. Combination of *lsm-2 *or *lsm-4 *RNAi with a mutation in any one of four different Class A genes caused a Muv phenotype, ranging from 20% to 96% of the animals at 25°C (Table 2). Genetic epistasis analysis revealed that this splice factor associated synMuv B phenotype depends on a functional *let-60 *Ras signaling pathway and is reduced in *lin-3 *EGF mutants, in agreement with genetic epistasis analysis of other synMuv B genes (Table 2) [9,50].

**Table 2:** Novel synMuv B genes and genetic epistasis analysis.

	**% of animals with 2, 3, 4 or 5 pseudovulvae**					
	**2**	**3**	**4**	**5**	**% of Muv**	**n**
	
**synMuv B activity**						
*rsr-2(RNAi)*	0%	0%	0%	0%	0%	445
*rsr-2(RNAi); lin-15A(n767)*	8.6%	1.3%	0%	0%	10%	453
*rsr-2(RNAi); lin-8(n111)*	1.5%	0%	0%	0%	2%	197
*rsr-2(RNAi); lin-38(n751)*	1.0%	0.1%	0%	0%	1%	787
*rsr-2(RNAi); lin-56(n2728)*	2.0%	0%	0%	0%	2%	595
						
*rrf-3(pk1426); lsm-2(RNAi)*	0%	0%	0%	0%	0%	>300
*lsm-2(RNAi); lin-15A(n767)*	1.5%	3.1%	1.0%	0%	6%	390
*lsm-2(RNAi) ****25°C***	0%	0%	0%	0%	0%	220
*lsm-2(RNAi); lin-15A(n767) ***25°C**	13.8%	42.5%	37.8%	0.5%	94%	188
*lsm-2(RNAi); lin-8(n111) ***25°C**	14.7%	9.5%	1.0%	1.0%	26%	136
*lsm-2(RNAi); lin-38(n751) ****25°C***	13.4%	27.4%	17.8%	0%	60%	157
*lsm-2(RNAi); lin-56(n2728) ****25°C***	7.1%	8.2%	4.7%	0%	20%	85
						
*rrf-3(pk1426); lsm-4(RNAi)*	0%	0%	0%	0%	0%	>300
*lsm-4(RNAi); lin-15A(n767)*	2.4%	5.0%	0.5%	0%	8%	420
*lsm-4(RNAi)****25°C***	0%	0%	0%	0%	0%	149
*lsm-4(RNAi); lin-15A(n767) ***25°C**	16.0%	40.8%	38.3%	0.6%	95%	162
*lsm-4(RNAi); lin-8(n111) ***25°C**	11.7%	9.0%	1.4%	0%	22%	145
*lsm-4(RNAi); lin-38(n751)****25°C***	10.5%	45.3%	23.2%	0%	79%	95
*lsm-4(RNAi); lin-56(n2728) ****25°C***	13.2%	14.0%	5.3%	0%	33%	114
						
*lin-37(RNAi); lin-15A(n767)*	27.6%	41.4%	24.1%	6.9%	100%	29
*lin-35(RNAi); lin-15A(n767)*	0%	27.5%	62.5%	10.0%	100%	40
*lin-35(RNAi); lin-38(n751)*	1.6%	1.6%	67.2%	22.9%	93%	61
*lin-35(RNAi); lin-15A(n767) ****25°C***	0%	0%	33.7%	58.7%	92%	92
*lin-35(RNAi); lin-8(n111) ****25°C***	1.2%	7.1%	29.4%	0%	37%	85
*lin-35(RNAi); lin-56(n2728) ****25°C***	1.2%	6.1%	37.8%	45.1%	89%	82
						
*snr-1(RNAi)(L1)(MV)*	0%	0%	0%	0%	0%	>200
*lin-35(n2239); snr-1(RNAi)(L1)(MV)*	0%	0%	0%	0%	0%	>200
*snr-1(RNAi)(L1)(MV); lin-15A(n767)*	9.3%	3.6%	4.3%	0.4%	18%	278
*snr-1(RNAi)(L1)(JA); lin-15A(n767)*	1.6%	3.2%	0%	0%	5%	62
*snr-1(RNAi)(L1)(MV); lin-56(n2728)*	2.4%	1.4%	0.7%	0%	5%	293
*snr-1(RNAi)(L1)(MV); lin-38(n751)*	3.3%	1.1%	0%	0%	4%	132
*snr-2(RNAi)(L1)(MV); lin-15A(n767)*	1.0%	1.0%	0%	0%	2%	93
*snr-3(RNAi)(L1)(MV)*	0%	0%	0%	0%	0%	>200
*lin-35(n2239); snr-3(RNAi)(L1)(MV)*	0%	0%	0%	0%	0%	>200
*snr-3(RNAi)(L1)(MV); lin-15A(n767)*	14.0%	4.0%	0.5%	0%	19%	221
*snr-3(RNAi)(L1)(MV); lin-38(n751)*	2.4%	0.8%	0%	0%	3%	126
*snr-4(RNAi)(L1)(MV); lin-15A(n767)*	9.7%	4.8%	1.2%	0%	16%	83
*snr-4(RNAi)(L1)(JA); lin-15A(n767)*	7.1%	3.2%	0%	0%	10%	156
*snr-5(RNAi)(L1)(MV); lin-15A(n767)*	1.3%	1.3%	0.6%	0%	3%	158
*snr-6(RNAi)(L1)(MV); lin-15A(n767)*	4.7%	3.5%	0%	0%	8%	257
*snr-7(RNAi)(L1)(MV); lin-15A(n767)*	10.7%	7.1%	2.2%	0%	20%	140
						
						
**EGF/RAS/MAPK epistasis**						
*rsr-2(RNAi); let-23(sy97); lin-15A(n767)*	0%	0%	0%	0%	0%	99
*rsr-2(RNAi); let-60(n1876); lin-15A(n767)*	0%	0%	0%	0%	0%	43
*rsr-2(RNAi); lin-3(n378); lin-15A(n767)*	5.0%	1.3%	0%	0%	6%	80
						
*let-60(n1876); lsm-2(RNAi); lin-15A(n767)*	0%	0%	0%	0%	0%	34
*lin-3(n378); lsm-2(RNAi); lin-15A(n767)*	1.6%	0%	0%	0%	2%	250
						
*lsm-4(RNAi); let-60(n1876); lin-15A(n767)*	0%	0%	0%	0%	0%	64
*lsm-4(RNAi); lin-3(n378); lin-15A(n767)*	2.7%	0.6%	0%	0%	3%	180
						
*lin-35(RNAi); let-23(sy97); lin-15A(n767)*	0%	0%	0%	0%	0%	58
*lin-35(RNAi); let-60(n1876); lin-15A(n767)*	0%	0%	0%	0%	0%	25
*lin-35(RNAi); lin-3(n378); lin-15A(n767)*	3.2%	15.9%	74.6%	6.3%	100%	63

RNAi experiments were performed at 20°C on young adults unless indicated otherwise. Multivulva animals were scored in the next generation after 5, 7 and 9 days. All synMuv A mutants were 0% Muv at 20°C and 25°C. (L1): feeding RNAi started at first larval stage. (JA): clone from RNAi library generated by the Ahringer lab. (MV): clone from RNAi library generated by the Vidal lab.

**Figure 4:**
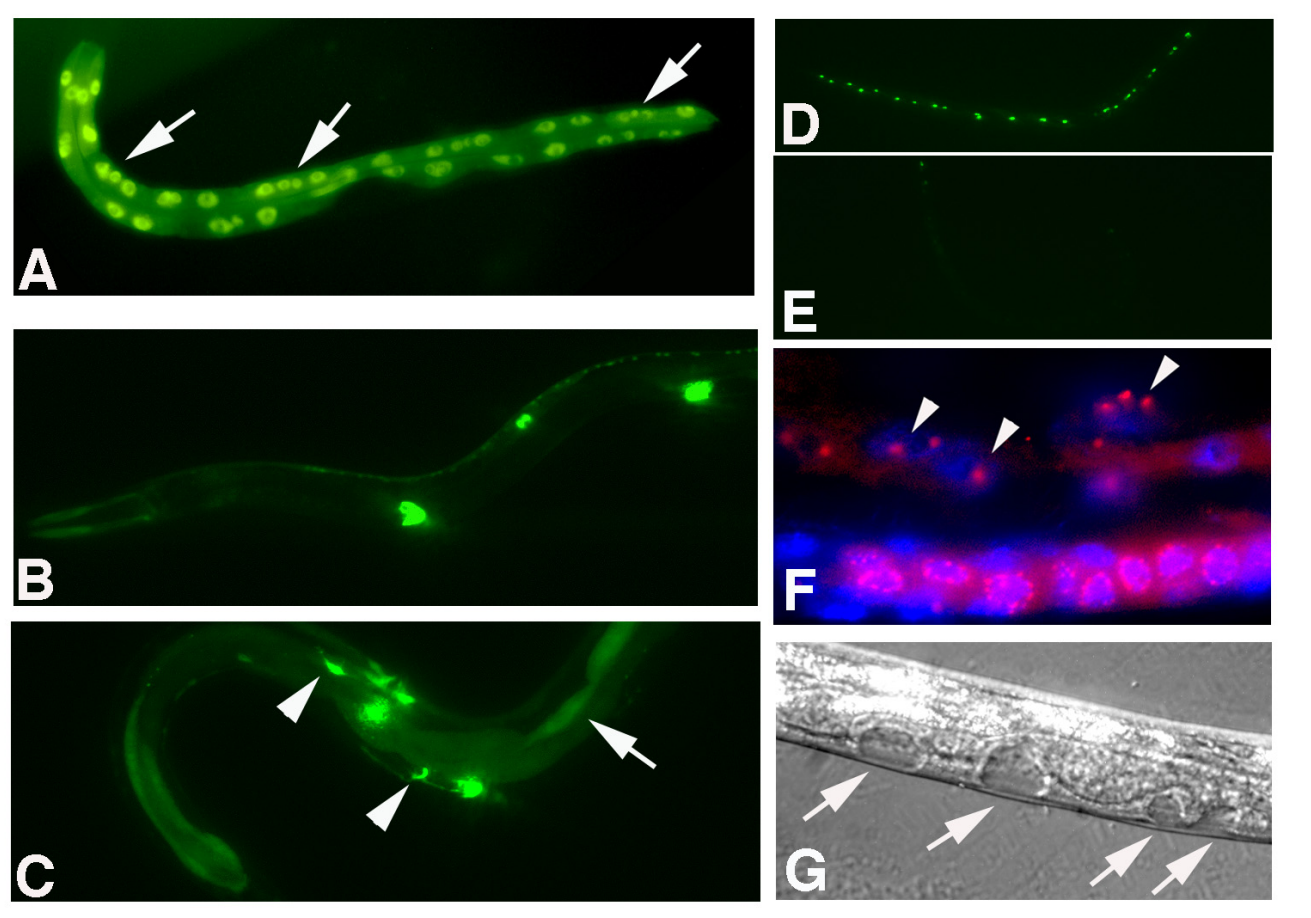
**Inactivation of specific spliceosome components resembles synMuv B genes**. (A) *rsr-2 *RNAi by feeding in WT (N2) animals results in extra intestinal nuclei (arrows) (this animal: 41 nuclei), as visualized by intestine specific expression of *elt-2::GFP *(B) *lag-2*::GFP reporter shows expression in distal tip cells and vulval cells (C) Ectopic expression of *lag-2*::GFP reporter in the intestine (arrows) and other cells (arrowheads) after *lsm-2 *RNAi. (*D*) Expression of *scm*::GFP in seam cells is not affected by *lsm-4 *RNAi. (E) *lin-35 *RNAi induced silencing of the *scm*::GFP transgene. (F) *snr-3 *RNAi by feeding in WT animal induced ectopic P granules in somatic cells (arrowheads), in addition to germ cell precursor (bottom). DAPI staining of DNA is in blue, staining of P granules detected with the K76 antibody in red. (G) Induction of ectopic vulval structures following *snr-4 *RNAi in *lin-15A *mutant larvae (L3 stage).

In addition to their role in vulval-fate determination, synMuv B genes show specific combinations of other loss-of-function characteristics that include: deregulation of cell-cycle entry, ectopic expression or silencing of transgenes, RNAi hypersensitivity and expression of germline P granules in the soma [26,51]. Although the ten splicing-related synMuv B genes are essential for viability, partial inactivation by feeding RNAi induced other properties of synMuv B genes (Table 3). For example, like *lin-35 *inactivation (see above), *rsr-2 *RNAi led to formation of extra intestinal nuclei (41.3 ± 3.6 (n = 37) versus control: 33.3 ± 1.9 (n = 27) in the *elt-2*::GFP strain) (Figure 3). In addition, RNAi of several synMuv B genes induces ectopic expression of a *lag-2::*GFP reporter in the intestine [51] and we observed the same phenotype after RNAi of *lsm-2 *and *lsm-4*, a weaker phenotype after RNAi of *snr *genes, but no effect after *rsr-2 *RNAi (Table 3). Moreover, like *lin-35 *loss of function, feeding RNAi of *snr-2 *and *snr-3 *induced ectopic P-granules in the soma (Figure 4). *lin-35 *RNAi also causes silencing of the *scm::*GFP transgene and partial rescue of *cyd-1 *larval arrest. These latter effects were not observed after RNAi of the ten novel synMuv B genes. Together, the observed effects suggest that components of the general splicing machinery contribute surprisingly specific functions to some, but not all, processes mediated by *lin-35 *Rb and other synMuv B genes (Table 3).

**Table 3:** Summary of RNAi phenotypes of splicing related synMuv genes in synMuv B activity, transgene silencing (*scm::*GFP), ectopic expression of *lag-2::GFP *reporter, extra intestinal cells (*elt-2::GFP*), ectopic P granules, and rescue of *cyd-1*.

	***lin-35***	***rsr-2***	***lsm-2***	***lsm-4***	***snr's *(1–7)**
**RNAi phenotype in WT**	Rbs	Ste, Lva	WT	WT	Emb, Lva, Lvl
**synMuv B**	+++	+	+	+	+
**synMuv B (25°C)**	+++	n/a	++	++	n/a
**Transgene silencing (25°C)**	++	-	-	-	n/a
**Ectopic *lag-2::GFP***	+	-	-	-	+
**Ectopic *lag-2::GFP *(25°C)**	++	-	++	++	+
**Extra intestinal cells**	+	+	-	-	-
**Ectopic P granules**	+++	-	-	-	+
***cyd-1 *rescue**	+	-	-	-	-

Summary of RNAi phenotypes of splicing related genes: synMuv B activity, transgene silencing (scm::GFP), ectopic expression of lag-2::GFP reporter, extra intestinal cells (elt-2::GFP), ectopic P granules, and rescue of cyd-1. If 25°C is not indicated, experiments were performed at 20°C. “+++”, “++”, “+” and “–“stand for strong, moderate, low and no effect respectively. (n/a: not annotated).

How can spliceosome components behave as synMuv B genes? Evidently, complete inactivation of splicing should cause loss-of-function of all intron-containing genes, including synMuv B genes. However, we used partial loss-of-function conditions that should not completely block splicing, and three of the phenotypic readouts involve increased, rather than reduced, expression levels. Specifically, the synMuv phenotype likely depends on increased expression of *lin-3 *EGF in the hyp7 hypodermal syncytium [50], extra nuclear divisions require induction of S phase genes and ectopic *lag-2 *expression needs *de novo *expression of the transgene reporter. Importantly, RNAi for many other critical splicing components did not cause a Multivulva phenotype (see Additional file 2, Supplementary Table 2B), indicating that this phenotype is not a consequence of a general shutdown of the splicing process. A more specific defect in splicing of synMuv B pre-mRNAs cannot be excluded. However, it is unclear why splicing of one or more synMuv B messages would depend strongly on a subset of general splicing factors, and not on many others. Moreover, because the splicing related genes display non-overlapping synMuv B features, a single synMuv B pre-mRNA that is particularly sensitive to (alternative) splicing does not provide an explanation for the observed phenotypes (Table 3).

Functions independent from splicing have been reported for Sm and Sm-like (LSM) proteins and should be considered as alternative explanations [49,52-54]. Studies in *C. elegans *have shown that Sm proteins are components of germline specific P granules and regulate multiple aspects of the germ-cell fate [49,52]. While LSM proteins were not examined, these studies indicated that Sm ribonucleoprotein complexes can act distinct from pre-mRNA splicing, possibly in the posttranscriptional control of maternal mRNAs. Recently, cytoplasmic structures known as P bodies or GW bodies have been linked to mRNA degradation and storage [53]. Yeast and human LSM 1–7 proteins have been shown to localize to these cytoplasmic bodies and have been implicated in the degradation of mRNA [54]. Thus, the synMuv B activity of specific splicing related proteins could relate to alternative functions in mRNA metabolism and translation. Such functions could inhibit gene expression post-transcriptionally, in concert with transcriptional repression mediated by LIN-35 Rb complexes. If this model is correct, it could indicate a surprising overlap between repression of gene expression at the transcriptional level in the nucleus and post-transcriptional level in the cytoplasm.

Of great interest, a link between mRNA processing bodies and the microRNA (miRNA) and RNAi pathways has recently been established [55-57]. In addition, RNA-interference related pathways have been observed to cooperate with *lin-35 *Rb [19], and Sm proteins have also been found to interact with the RNAi machinery in *C. elegans *[58,59]. Specifically: while *snr-7 *is required for RNAi and transgene expression [58], SNR-3 physically interacts with DCR-1, a key enzyme in the RNAi and miRNA pathways [59]. Like *lin-35 *and *rsr-2 *(this study), *dcr-1 *also negatively regulates nuclear divisions in the intestine [19]. Thus, cooperation between LIN-35 Rb and Sm/LSm protein functions might involve miRNA/siRNA pathways.

**Figure 5:**
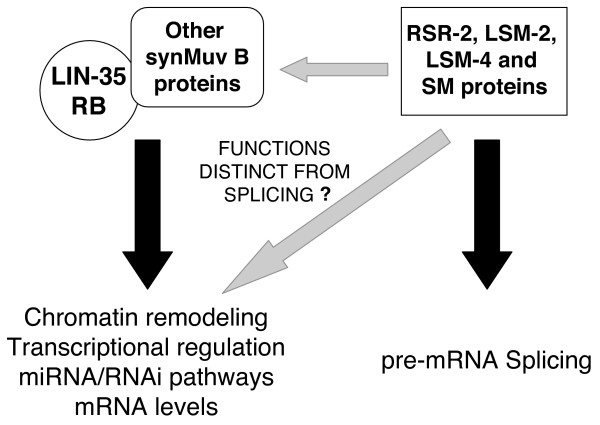
**Novel roles of specific spliceosome components in the synMuv B pathway**. RSR-2, LSM-2, LSM-4 and SM proteins may have functions that reduce gene expression and are independent of pre-mRNA splicing. The phenotypic overlap with *lin-35 *Rb mutants may indicate functions in transcription repression, chromatin modification, miRNA/RNAi pathway modulation or mRNA metabolism.

Further studies will be needed to examine potential coupling between transcription, splicing and RNAi-related processes. Based on the overlapping loss-of-function phenotypes, we propose a model in which specific splicing components cooperate with *lin-35 *synMuv B complexes in repression of gene expression, either by promoting mRNA turnover/translation inhibition or by taking part in multi-protein complexes that regulate transcription, chromatin remodeling, and/or miRNA/RNAi mediated post-transcriptional gene silencing (Figure 5).

## Conclusion

Our results provide an extensive series of candidate *lin-35 *interacting genes, validate *zfp-2 *as a novel *lin-35 *synthetic lethal gene, and implicate a subset of spliceosome components in gene regulation in conjunction with the *lin-35 *Rb pathway.

## Methods

### Strains

We used the wild-type strain Bristol N2 and the following mutations:

LG I: *lin-35*(*n745, n2239*). LG II: *rrf-3*(*pk1426*), *zfp-2*(*tm557*), *let-23*(*sy97*). LG III: *lin-37*(*n758*), *hpl-2*(*ok917*). LG IV: *eri-1*(*mg366*), *let-60*(*n1876*), *unc-22*(*s7*), *lin-3*(*n378*). LG X: *lin-15*(*n744*), *lin-15A*(*n767*)

Integrated arrays: *rtIs14 *[*elt-2*::GFP; *osm-10*::HT150Q] IV (a gift from P. W. Faber and A. Hart). *ezIs2 *[*fkh-6*::GFP + *unc-119*(+)] [39]. In addition, we used the integrated array *ccIs4251 *[*myo-3*::Ngfp-lacZ, pSAK4(*myo-3*::Mtgfp)], mapped to LGI (+2.5), to balance *lin-35 *(*n2239*) in strain *lin-35*(*n2239*)/*ccIs4251 *I, *zfp-2*(*tm557*) II.

### RNAi screens

RNAi experiments were performed as detailed in Rual et al. [25]. In the screen, dsRNA was delivered by feeding L1 synchronized animals on agar plates containing IPTG (6 mM) plus ampicillin and tetracycline. 10,953 RNAi clones were tested in 24-well plates. In all RNAi experiments, wild-type animals and *lin-35 *mutants were simultaneously examined in duplicate (thus, 6 different genes were tested per 24-well plate). RNAi phenotypes were scored visually on days 4 and 6 after L1 worms were put to culture. Because of a substantial variation in feeding RNAi phenotypes, the large number of genes tested and range of phenotypes scored, quantitative determination of phenotypes was unpractical. Therefore, candidates were selected based on obvious and reproducibly stronger phenotypes in *lin-35 *mutants, as compared to other backgrounds described in the text. For all genes of interest, inserts of feeding RNAi vectors were checked by PCR amplification and DNA sequence analysis.

For many genes, a low penetrant RNAi phenotype was observed that became much stronger when combined with inactivation of *lin-35 *Rb. We refer to such phenotypes as "enhanced", rather than "synthetic", in combination with *lin-35*. Lethal (Let) is defined as animals unable to produce viable progeny. When possible, a more informative description is used: Emb (embryonic lethal), Lvl (larval lethal), Lva (larval arrest), or Ste (sterile). Other phenotypes scored were: Unc (uncoordinated), Prl (paralyzed), Dpy (dumpy), Bmd (body morphology defective), Sck (sick), Bli (blistered), Mlt (molting defective), Slm (slim), Him (high incidence of males), Pvl (protruding vulva), Muv (multivulva), Clr (clear), Slu (sluggish), Lon (long), Sma (small), Gro (growth rate abnormal), Egl (egg laying defective), Rbs (reduced brood size), Ooc (oocyte formation abnormal), Stp (sterile progeny), and Rup (ruptured). See Additional file 1, Supplementary Table 1, for further information and details.

To screen for synMuv B genes among splicing-related genes, we performed feeding RNAi starting from L4 and L1 stage animals.

### dsRNA injection

Preparation of dsRNA for injection: DNA templates were amplified by PCR from L4440 plasmids using the following primers:

pL440-dest-RNAi-FOR (5'GTTTTCCCAGTCACGACGTT3') and pL440-dest-RNAi-REV (5'TGGATAACCGTATTACCGCC3'). PCR products were used as the templates for synthesis of dsRNA with an *in vitro *transcription kit (T3 T7 Ambion). The RNA were incubated at 72°C for 10 minutes followed by 30 minutes at 37°C to allow annealing. The quality and amount of dsRNA was examined by electrophoresis. For injection, we used dsRNA at a concentration of 0.5 to 2 μg/μl.

### Immunostaining

Worm larvae were permeabilized on coverslips using freeze/cracking. Samples were fixed in ice-cold methanol (10 min.) followed by ice-cold acetone (10 min.). K76 mouse monoclonal antibodies were used (1:2) to detect P granules. Rhodamine-conjugated anti-mouse antibodies were used as secondary antibodies (1:200).

For staining of DNA, animals were fixed in Carnoys fixative at 4°C for 24 hours. After rehydration and washes, samples were incubated for 10 minutes in a solution of 1 μg/ml DAPI.

### Analysis of *zfp-2 *expression

*zfp-2*::GFP transcriptional fusions were generated by *in vitro*recombination using the promoterome clones and the destination vector pdest-DD03 as described in Dupuy et al. [61]. Clone and primers sequences can be retrieved from Promoterome database [67].

## Authors' contributions

JC conducted most of the experiments. JFR and MV generated and provided the RNAi library. AC participated in some of the RNAi screens. DD generated the *zfp-2 *expression vector. SV conceived the study, and participated in its design and coordination. JC and SV wrote the manuscript. All authors have read and approved the final manuscript.

## Supplementary Material

Additional File 1 Supplementary Table 1. Results from RNAi screens and functional categories of candidates genes.  (additional legend : Additional file 6) Click here for file

Additional File 3 Figure S1. 14 genes previously shown to interact with *lin-35*. Click here for file

Additional File 4 Figure S2. *zfp-2 *gene structure and KRAB domain conserved sequence. Click here for file

Additional File 5 Figure S3. *zfp-2*, as *lin-35*, is required for expression of extrachromosomal arrays. Click here for file

Additional File 2 Supplementary Table 2. Splicing related genes and associated RNAi phenotypes. Click here for file

Additional File 6 Additional legends. Additional legends for additional files 1 and 2. Click here for file
