# Supplementary material for: Large-scale RNAi screens identify novel genes that interact with the C. elegans retinoblastoma pathway as well as splicing-related components with synMuv B activity
Source: BMC Dev Biol. 2007 Apr 6;7:30. doi: 10.1186/1471-213X-7-30 (PMC1863419; doi:10.1186/1471-213X-7-30)
Supplement: Additional File 3 — Figure S1. 14 genes previously shown to interact with lin-35. [file 1471-213X-7-30-S3.pdf]

Additional file 3

Gene	Class	Reference
ubc-18	Protein degradation	Fay et al, 2003
psa-1	Protein degradation	Cui et al, 2004
lin-23	Protein degradation	Fay et al, 2001
rde-1	RNA-related	Grishok and Sharp, 2005
rde-4	RNA-related	Grishok and Sharp, 2005
cki-1	Cell cycle / Signaling	Boxem and van den Heuvel, 2001
fzr-1	Cell cycle / Signaling	Fay et al, 2001
cdc-14	Cell cycle / Signaling	Saito et al, 2004
pha-1	Transcription, Replication and Chromatin	Fay et al, 2004
xnp-1	Transcription, Replication and Chromatin	Cardoso et al, 2005; Bender et al, 2004
him-17	Transcription, Replication and Chromatin	Reddy and Villeneuve, 2004
gon-14	Transcription, Replication and Chromatin	Chesney et al, 2006
lin-8	Transcription, Replication and Chromatin	Davison et al, 2005
lin-15A	Unknown	Clark et al, 1994

14 genes previously shown to interact with *lin-35*
