# Supplementary material for: Large-scale RNAi screens identify novel genes that interact with the C. elegans retinoblastoma pathway as well as splicing-related components with synMuv B activity
Source: BMC Dev Biol. 2007 Apr 6;7:30. doi: 10.1186/1471-213X-7-30 (PMC1863419; doi:10.1186/1471-213X-7-30)
Supplement: Additional File 4 — Figure S2. zfp-2 gene structure and KRAB domain conserved sequence. [file 1471-213X-7-30-S4.pdf]

Additional file 4

A

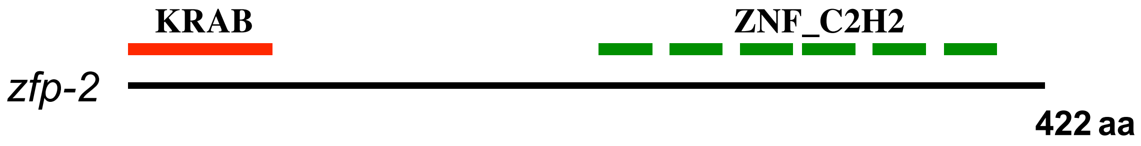

B

Consensus KRAB A DOMAIN RTLVTFKDVFVDFTREEWKLLDTAQQIVYRNVMLENYKLLVSL

Human RBaK MNTLQGPVSFKDVAVDFTQEEWQQLDPDEKITYRDVMLENYSHLVSV

C. elegans zfp-2 MEEMMMNDPSAMVIYEEEVTTAPNLPCSLIQQRSWDEEKPIGY

C. briggsae homolog MEELMLEDPNATVLYEEEVTTATDISTDIPSTSRAWSNEKPLG

(A) *zfp-2* encodes a protein containing six C2H2 zinc fingers and a putative KRAB motif. (B) Homology among a consensus KRAB domain, the KRAB domain for human RBak (interacts with Rb), and the putative KRAB domain of *zfp-2* and its homolog in *C. briggsae*.
