# Supplementary material for: Large-scale RNAi screens identify novel genes that interact with the C. elegans retinoblastoma pathway as well as splicing-related components with synMuv B activity
Source: BMC Dev Biol. 2007 Apr 6;7:30. doi: 10.1186/1471-213X-7-30 (PMC1863419; doi:10.1186/1471-213X-7-30)
Supplement: Additional File 5 — Figure S3. zfp-2, as lin-35, is required for expression of extrachromosomal arrays. [file 1471-213X-7-30-S5.pdf]

Additional file 5

	% of rollers	n
tnIs6[lim-7::GFP + rol-6(su1006)]	84%	373
tnIs6[lim-7::GFP + rol-6(su1006)]; zfp-2(RNAi)	58%	663
tnIs6[lim-7::GFP + rol-6(su1006)]; lin-35(RNAi)	8%	525
tnIs6[lim-7::GFP + rol-6(su1006)]; lin-35(RNAi); zfp-2(RNAi)	2%	399

zfp-2, as _lin-35_, is required for expression of extrachromosomal arrays

RNAi was performed by injection of dsRNA. “n” indicates number of animals (F1) scored, after injecting dsRNA in 3 to 7 hermaphrodites for each experiment. As control, *lin-15A*; *lin-35* RNAi; *zfp-2* RNAi F1 animals were Muv and Sterile.
