# Supplementary material for: Large-scale RNAi screens identify novel genes that interact with the C. elegans retinoblastoma pathway as well as splicing-related components with synMuv B activity
Source: BMC Dev Biol. 2007 Apr 6;7:30. doi: 10.1186/1471-213X-7-30 (PMC1863419; doi:10.1186/1471-213X-7-30)
Supplement: Additional File 6 — Additional legends. Additional legends for additional files 1 and 2. [file 1471-213X-7-30-S6.pdf]

Additional file 6

Additional files 1 and 2 Legends

Additional file 1. Supplementary Table 1 (Excel File)

Worksheet A. Results from the primary and secondary large-scale feeding RNAi screens. For 523 genes, synthetic or enhanced RNAi phenotypes were observed in *lin-35(n2239)* mutant animals as compared to WT (N2). Subsequently, 244 candidate synthetic interacting genes were selected in a secondary screen that included *rrf-3* mutants. Note: these analyses also include the first time description of an RNAi phenotype for 69 genes. Lethal is defined as animals unable to produce viable progeny and includes the Emb, Lvl, Lva, Let, and Ste classes.

Worksheet B. Results from feeding RNAi experiments for 244 genes, assayed in various RNAi hypersensitive strains.
Gene information was retrieved from WS153 [66].
Abbreviations were used for phenotypes as described in Rual et al, 2004 [25], see also Material and Methods. Lva (larval arrest), Unc (uncoordinated), Prl (paralyzed), Dpy (dumpy), Bmd (body morphology defective), Sck (sick), Bli (blistered), Mlt (molting defect), Slm (slim), Him (high incidence of males), Pvl (protruding vulva), Muv (multivulva), Clr (clear), Slu (sluggish), Lon (long), Sma (small), Gro (growth rate abnormal), Egl (egg laying defective), Ste (sterile), Rbs (reduced brood size), Let (Lethal), Emb (embryonic lethal), Ooc (oocyte formation abnormal), Stp (sterile progeny), Rup (ruptured) and Lvl (larval lethal). When the phenotype is observed in the F1 generation, F1 is added before the phenotype name (i.e, F1 Lva). Phenotypes observed with partial penetrance are indicated with a "p", for example, pLva for partially penetrant larval lethality. A partial phenotype is scored when the phenotype is observed in <90% of the P_0 or F1 progeny. When the brood size was lower than 10 embryos per animal, highly reduced brood size is indicated (high Rbs).

Worksheet C. Functional categories and description of the 57 candidates genes.

Additional file 2. Supplementary Table 2 (Excel file).

Worksheet A. List of 135 splicing related genes in *C. elegans* resulted from BLAST searches of 145 human spliceosome-component sequences [S1] against version WS148 of the worm genome. Worksheet B. RNAi phenotypes of 135 splicing related genes after feeding L1 and young adults of *lin-15A* mutants.

S1 Zhou, Z., Licklider, L.J., Gygi, S.P., and Reed, R. (2002). Comprehensive proteomic analysis of the human spliceosome. Nature 419, 182-185.
